# Sparsity and locally low rank regularization for MR fingerprinting

**DOI:** 10.1002/mrm.27665

**Published:** 2019-02-05

**Authors:** Gastão Lima da Cruz, Aurélien Bustin, Oliver Jaubert, Torben Schneider, René M. Botnar, Claudia Prieto

**Affiliations:** ^1^ King’s College London School of Biomedical Engineering and Imaging Sciences London United Kingdom; ^2^ Philips Healthcare Guilford United Kingdom; ^3^ Pontificia Universidad Católica de Chile Escuela de Ingeniería Santiago Chile

**Keywords:** compressed sensing, locally low rank, low rank, MR fingerprinting, quantitative MRI

## Abstract

**Purpose:**

Develop a sparse and locally low rank (LLR) regularized reconstruction to accelerate MR fingerprinting (MRF).

**Methods:**

Recent works have introduced low rank reconstructions to MRF, based on temporal compression operators learned from the MRF dictionary. In other MR applications, LLR regularization has been introduced to exploit temporal redundancy in local regions of the image. Here, we propose to include spatial sparsity and LLR regularization terms in the MRF reconstruction. This approach, so called SLLR‐MRF, further reduces aliasing in the time‐point images and enables higher acceleration factors. The proposed approach was evaluated in simulations, T_1_/T_2_ phantom acquisition, and in vivo brain acquisitions in 5 healthy subjects with different undersampling factors. Acceleration was also used in vivo to enable acquisitions with higher in‐plane spatial resolution in comparable scan time.

**Results:**

Simulations, phantom, and in vivo results show that low rank MRF reconstructions with high acceleration factors (<875 time‐point images, 1 radial spoke per time‐point) have residual aliasing artifacts that propagate into the parametric maps. The artifacts are reduced with the proposed SLLR‐MRF resulting in considerable improvements in precision, without changes in accuracy. In vivo results show improved parametric maps for the proposed SLLR‐MRF, potentially enabling MRF acquisitions with 1 radial spoke per time‐point in approximately 2.6 s (~600 time‐point images) for 2 × 2 mm and 9.6 s (1750 time‐point images) for 1 × 1 mm in‐plane resolution.

**Conclusion:**

The proposed SLLR‐MRF reconstruction further improves parametric map quality compared with low rank MRF, enabling shorter scan times and/or increased spatial resolution.

## 1 INTRODUCTION

MR fingerprinting (MRF)^1^ is a novel transient state relaxometry framework that provides simultaneous multiparametric maps. In MRF, sequence parameters such as flip angle (FA) and repetition time (TR) are varied throughout the acquisition of several (in the order of thousand) time‐point images, such that different tissues experience different magnetization evolutions. These tissue specific signal evolutions (fingerprints) can be matched in a voxel‐by‐voxel basis to a previously simulated set of signals (dictionary) to identify the underlying tissue parameters (e.g., T_1_, T_2_). Non‐Cartesian sampling trajectories are usually used to reduce scan time in MRF, under the premise that residual aliasing artifacts mimic pseudorandom noise in each voxel fingerprint. Nevertheless, if the aliasing is not spatiotemporally incoherent or the aliasing is too severe, these errors may propagate from the time‐point images into the parametric maps.^2^

Recent works have focused on developing MRF tailored reconstructions to further improve parametric map quality and/or reduce scan time. Frameworks incorporating compressed sensing,^3^ simultaneous multislice,^4^, ^5^, ^6^ iterative multiscale,^7^ and sliding window^8^, ^9^, ^10^ reconstruction have been proposed. General solutions for data sharing in MRF^2^, ^11^, ^12^, ^13^, ^14^ and other parameter mapping techniques^15^, ^16^ using low rank approximations have also been introduced. Correlations between fingerprint evolutions (and thus between time‐point images) can be exploited to project the data into a temporally compressed domain with superior sampling properties (in the Nyquist sense). Consequently, reconstruction of temporally compressed images (low rank approximation of the MRF time‐point images) is faster and better posed than reconstructing each time‐point image separately. The performance of these low rank approximation methods can be further improved with regularization, as proposed Assländer et al^13^ and Hamilton et al^17^ which use dictionary match regularization and wavelet sparsity terms, respectively. In MRI applications like dynamic imaging and T_2_ mapping, locally low rank (LLR) constraints have been shown to be powerful regularizers.^16^, ^18^, ^19^, ^20^ Local image blocks have a smaller rank than complete images.^20^ This corresponds to a higher level of data redundancy, which can be leveraged to reduce noise and aliasing artifacts.

In this work, we propose to include sparsity and LLR regularization terms in the low rank MRF framework. Spatial sparsity is enforced in the wavelet domain and LLR is enforced in the temporally compressed domain of the low rank approximation. The proposed sparse and LLR regularized MRF (SLLR‐MRF) approach was evaluated in simulations, standardized T_1_/T_2_ phantom acquisitions, and in vivo brain acquisitions in 5 healthy subjects.

## 2 METHODS

### 2.1 Low rank approximation of the time‐point MRF images

MRF is designed to lead the magnetization through a continuous transient state evolution. Different tissues experience distinct signal evolutions; however, the magnetization evolutions are correlated in time, according to the underlying sequence parameters. Thus, the sequence of *N_t_* (~1000) time‐point MRF images can be temporally compressed to *N_s_* (~10) singular images (*N_s_ < N_t_*). The MRF reconstruction of *N_s_* singular images is faster (less images) and better conditioned (more data per image) than reconstructing individual time‐points. As proposed in McGivney et al,^11^ a singular value decomposition (SVD) of the MRF dictionary D may be used to determine a global low rank approximation of the MRF signals: $D=U\Sigma V^{H}$. The temporal compression operator $U_{r}$ follows from a truncation (to the appropriate rank *r*) of the matrix U. This global low rank constraint may be enforced directly in the encoding operator $E=\mathrm{AFC}U_{r}$, where C are the coil sensitivities,^21^ F is the Fourier transform and A is the sampling operator.

In McGivney et al,^11^ similar results have been obtained reconstructing MRF with $FU_{r}$ or $U_{r}F$. F and $U_{r}$ have been shown to commute^13^ and similarly C and $U_{r}$ can also be shown to commute. Thus, the encoding operator may be re‐written as $E=AU_{r}FC$, which is beneficial because only *N_s_* Fourier transforms are needed instead of *N_t_*.^13^ Compression of k‐space using an MRF dictionary derived operator ($U_{r}$) has been investigated recently in several works.^2^, ^11^, ^13^ Because k‐space data at each time‐point are a linear combination of all imaged tissues, $U_{r}$ is a valid projection matrix (as long as the dictionary D accurately characterizes the acquisition and the expected tissues). The global low rank MRF reconstruction may be cast as a linear inverse problem^12^:

(1) $$\hat{x}=argmin_{x}\frac{1}{2}\;\;||{\mathrm{AU}}_{r}\mathrm{FCx}-k{\left|\right|}_{2}^{2}$$

where $\hat{x}$ are the singular images, and k are the acquired k‐space data. In the formulation above, **𝒙** ∈ $C^{N_{s}N_{n}}$, $C\in C^{N_{s}N_{n}N_{c}\times N_{s}N_{n}}$, $F\in C^{N_{s}N_{n}N_{c}\times N_{s}N_{n}N_{c}}$, $U_{r}\in C^{N_{t}N_{n}N_{c}\times N_{s}N_{n}N_{c}}$, **𝑨** ∈ $C^{N_{t}N_{k}N_{c}\times N_{t}N_{n}N_{c}}$,$k\in C^{N_{t}N_{k}N_{c}}$; $N_{n}$ is the number of pixels, $N_{c}$ is the number of coils and $N_{k}$ is the number of k‐space points (per time‐point). The low rank MRF reconstruction can be solved with the conjugate gradient algorithm.^22^

### 2.2 Sparsity and LLR regularization

MR images are known to have sparse representations in certain domains. This information can be leveraged to regularize an otherwise ill‐posed reconstruction by means of minimization of an l_1_‐norm.^23^ The problem in Equation 1 tends to be increasingly ill‐posed with increasing rank of the singular image and, therefore, the high rank images usually contain residual aliasing. Compressed sensing can be used to reduce noise and undersampling artifacts. Here, we choose the wavelet transform to exploit sparsity in the spatial dimension, similar to what has been proposed for cardiac MRF previously.^17^

Locally low rank regularization^18^, ^20^ can further exploit existing temporal information (i.e., between singular images). Local image blocks have a higher degree of redundant information and correspondingly lower rank than entire images. This prior information can be imposed on the singular images by enforcing each image block to have a low rank structure: $\arg\min_{x}\sum_{b}rank\left(R_{b}x\right)$, where $R_{b}$ selects an image block around pixel b and reshapes the block into a local Casorati matrix. The solution to this problem is known to be NP‐hard, so the nuclear norm is commonly used as a surrogate functional for rank minimization. Thus, the proposed SLLR‐MRF reconstruction is given by:

(2) $$\hat{x}=argmin_{x}\frac{1}{2}\;\;\|{\mathrm{AU}}_{r}{\mathrm{FCx}-k\|}_{2}^{2}+\sum_{b}\lambda_{b}\|R_{b}{x\|}_{*}+\lambda_{w}{\left\|\mathrm{Wx}\right\|}_{1}$$

where $\lambda_{b}$ controls the strength of sparsity in the singular values of $R_{b}x$, ${\left\|\cdot \right\|}_{*}$ denotes the nuclear norm, $\lambda_{w}$ is the sparsity strength in the compressed spatial domain, W is the wavelet transform (chosen sparsity domain) and ${\left\|\cdot \right\|}_{1}$ denotes the l_1_‐norm. The above optimization is solved here using the alternating direction method of multipliers (ADMM).^24^ The optimization in Equation 2 can be re‐stated as the following constrained minimization:

(3) $$\begin{array}{c} \left(\hat{x},\;{\hat{y}}_{b},\;\hat{z}\right)=argmin_{x,y,z}\frac{1}{2}\;\|{\mathrm{AU}}_{r}{\mathrm{FCx}-k\|}_{2}^{2}+\sum_{b}\lambda_{b}\|y_{b}{\|}_{*}+\lambda_{w}{\left\|z\right\|}_{1}\\ \text{s.t.}\;R_{b}x=y_{b}\;\text{and}\;\mathrm{Wx}=z \end{array}$$

yielding the corresponding Lagrangian form:

(4) $$\begin{array}{c} L\left(x,\;y_{b},\;z\right)=\frac{1}{2}\;\;\|{\mathrm{AU}}_{r}{\mathrm{FCx}-k\|}_{2}^{2}+\sum_{b}\lambda_{b}\|y_{b}{\|}_{*}+\lambda_{w}\left\|z_{1}\right\|\\ +\sum_{b}Re\left(〈u_{b},\;R_{b}x-y_{b}〉\right)+\sum_{b}{\left\|\frac{\mu_{1}}{2}R_{b}x-y_{b}\right\|}_{2}^{2}\\ +Re\left(〈v,\;\mathrm{Wx}-z〉\right)+\frac{\mu_{2}}{2}{\left\|\mathrm{Wx}-z\right\|}_{2}^{2} \end{array}$$

where $\mu_{i}$ determines regularization strengths and $U_{b}$ and v are Lagrangian multipliers. Following the ADMM, a solution is found by iteratively optimizing the above functional with respect to x, then with respect to y, then with respect to z and then followed by updating the dual variables $U_{b}$ and v. This leads to the following set of sub problems:

(5) $$\begin{array}{c} x^{j+1}=\arg\min_{x}\frac{1}{2}{\left\|{\mathrm{AU}}_{r}\mathrm{FCx}-k\right\|}_{2}^{2}+\\ \sum_{b}\frac{\mu_{1}}{2}\|R_{b}x-y_{b}^{j}+\frac{u_{b}^{j}}{\mu_{1}}{\|}_{2}^{2}+\frac{\mu_{2}}{2}{\left\|\mathrm{Wx}-z^{j}+\frac{v^{j}}{\mu_{2}}\right\|}_{2}^{2} \end{array}$$

(6) $$y_{b}^{j+1}=\;{\mathrm{argmin}}_{y_{b}}\frac{\lambda_{b}}{\mu_{1}}\|y_{b}{\|}_{*}+\frac{1}{2}{\left\|R_{b}x^{j+1}+\frac{u_{b}^{j}}{\mu_{1}}-y_{b}\right\|}_{2}^{2}$$

(7) $$z^{j+1}=\;{\mathrm{argmin}}_{z}\frac{\lambda_{w}}{\mu_{2}}{\left\|z\right\|}_{1}+\frac{1}{2}{\left\|{\mathrm{Wx}}^{j+1}+\frac{v^{j}}{\mu_{2}}-z\right\|}_{2}^{2}$$

(8) $$U_{b}^{j+1}\;=\;U_{b}^{j}+{R_{b}x}^{j+1}-y_{b}^{j+1}$$

(9) $$V^{j+1}\;=\;V^{j}+Wx^{j+1}-z^{j+1}$$

where j is the iteration number. Equation 5 amounts to a Tikhonov regularized problem, where x can be solved for using efficient methods like the conjugate gradient.^22^ In the second problem (Equation 6), the solution can be found by means of (hard) singular value thresholding (SVT): $y_{b}^{j+1}={\mathrm{SVT}}_{\lambda_{b}/\mu_{1}}\left(R_{b}x^{j+1}+\frac{{U_{b}}^{j}}{\mu_{1}}\right)$.^25^ For each block b, the SVD of $R_{b}x^{j+1}+\frac{{U_{b}}^{j}}{\mu_{1}}$ is computed, truncated to the rank determined by $\lambda_{b}/\mu_{1}$ and stored in the corresponding location in $y_{b}^{j+1}$. Similarly, the solution to Equation 7 is given by soft thresholding (ST): $z^{j+1}={\mathrm{ST}}_{\lambda_{w}/\mu_{2}}\left(Wx^{j+1}+\frac{V^{j}}{\mu_{2}}\right)$. Finally, the updates of $U_{b}$ and v are performed according to Equations 8 and 9. A diagram with the main operations within each ADMM iteration of the proposed SLLR‐MRF is shown in Figure 1. The proposed reconstruction takes place in the singular value domain, which is a global low rank approximation of the (uncompressed) time‐point series. The local rank of the singular images is smaller than their global rank, which is exploited with the term $\lambda_{b}{\|R_{b}x\|}_{*}$. The singular images are also sparse in the wavelet domain, which is exploited with the term $\lambda_{w}{\left\|Wx\right\|}_{1}$.

**Figure 1:**
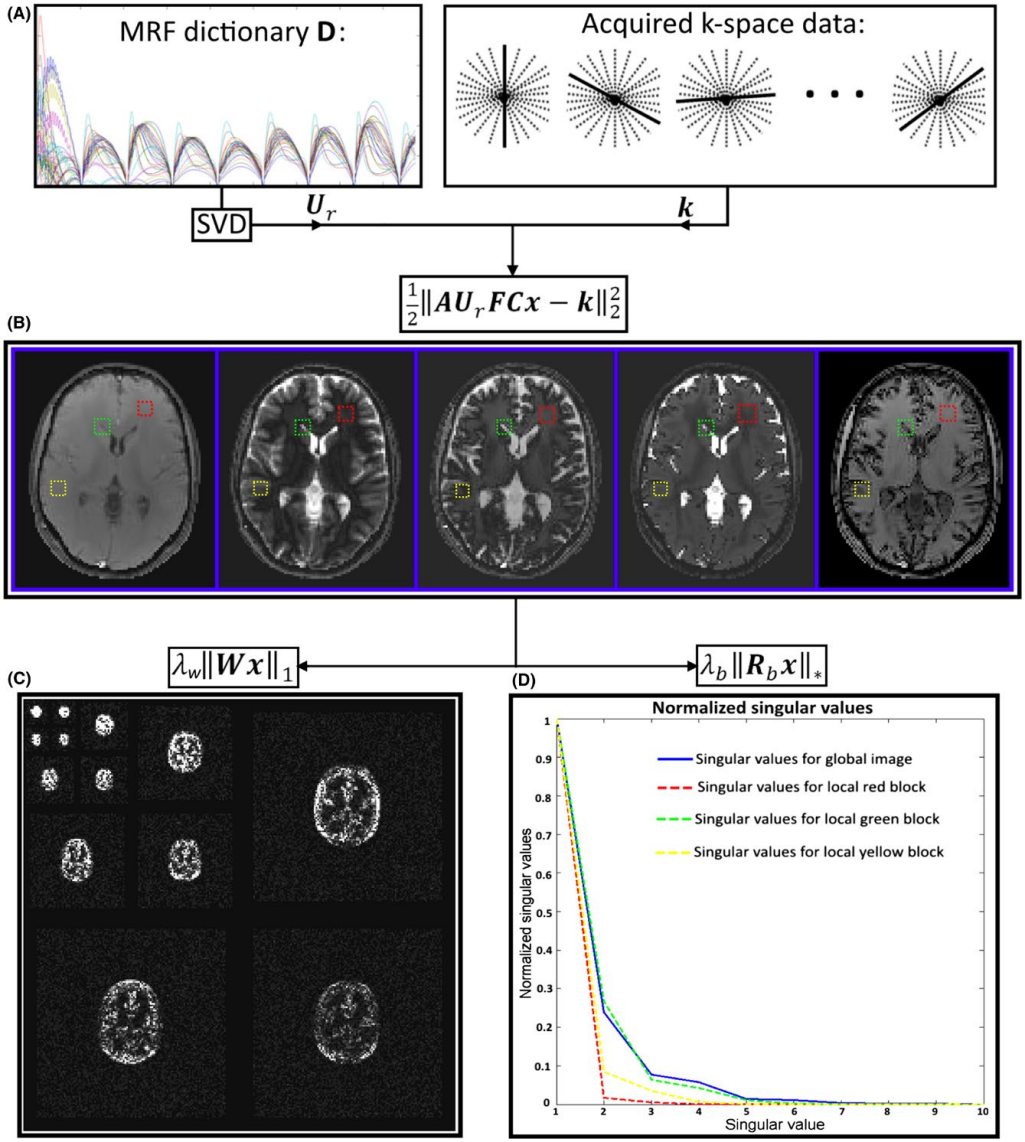
Diagram illustrating the main operations in 1 ADMM iteration of the proposed SLLR‐MRF. A, The precomputed MRF dictionary is used to derive the temporal compression operator $U_{r}$ by means of SVD. B, Global low rank is enforced by incorporating $U_{r}$ into the encoding operator, to reconstruct a temporally compressed time‐series x (also known as singular images). C, Spatial redundancy of the temporally compressed (singular) images is exploited in wavelet domain, which can be seen to be sparse. D, Temporal redundancy of the temporally compressed time‐series is exploited by means of LLR of image blocks (red, yellow, and green), where the normalized singular values decay faster than in the global case (blue)

### 2.3 Experiments

The proposed SLLR‐MRF was evaluated in simulations, phantom acquisitions, and in vivo brain data acquired on a 1.5T Ingenia MR system (Philips, Best, The Netherlands) using a 15‐element head coil. The study was approved by the institutional review board, and written informed consent was obtained from all subjects according to institutional guidelines.

### 2.4 Simulations

SLLR‐MRF was studied in a digital phantom based on in vivo brain data with realistic T_1_, T_2_, and M_0_ values. The simulation featured an acquisition similar to the one used in Jiang et al,^26^ except a fixed TR = 4.3 ms, 1750 time‐point images, golden radial trajectory^27^ with radial spoke per time‐point and slightly modified FA pattern (as the one used in a previous study^9^) were used. White Gaussian noise was added to the time‐point images with a standard deviation such that the signal‐to‐noise ratio (SNR) in the first time‐point image was approximately 20. The simulated k‐space was uniformly undersampled in time by keeping (1:*n*:1750) k‐space lines. Different simulated acquisition lengths were tested, with *n* = (1, 2, 3, 4), resulting in accelerated series with *N_t_* = (1750, 875, 584, 438) time‐points, respectively. Each series was reconstructed with both the low rank MRF approximation and the proposed SLLR‐MRF. To investigate the performance of sparsity and LLR constraints separately, each series was additionally reconstructed using only the sparse regularization (S‐MRF) and LLR regularization (LLR‐MRF). The normalized root mean square error (NRMSE) was measured on the reconstructed parametric maps with a mask excluding the cerebrospinal fluid (CSF), skull, and scalp of the digital phantom.^28^

### 2.5 Data acquisition

2D MRF acquisitions were performed on a standardized T_1_/T_2_ phantom^29^ and in 5 healthy subjects. The same sequence as in simulations was used for these acquisitions with the following relevant parameters: initial inversion recovery pulse, gradient echo readout, TE/TR = 1.23/4.3 ms, 1750 time‐points, golden radial spoke per time‐point, resolution = 2 × 2 mm^2^, field of view = 320 × 320 mm^2^, 10 mm slice thickness, total scan time = 7.5 s. For both the phantom and in vivo acquisitions, the acquired k‐space data were retrospectively undersampled in time (as previously described for the simulations) resulting in accelerated series with *N_t_* = (1750, 875, 584, 438) time‐points.

To investigate the use of SLLR‐MRF to enable higher in‐plane spatial resolution in comparable scan time than current reference (2 × 2 mm^2^ in‐plane resolution, 1750 time‐points) a second MRF acquisition was performed on the healthy subjects. High in‐plane spatial resolution data were acquired with the same parameters as in the previous in vivo experiment, except for TE/TR = 1.73 / 5.5 ms, in‐plane spatial resolution = 1 × 1 mm^2^, total scan time = 9.6 s.

### 2.6 Image reconstruction

The proposed SLLR‐MRF reconstruction was implemented off‐line in MATLAB (Mathworks, Natick, Massachusetts, USA). Coil sensitivity maps were estimated from the data itself using ESPIRiT.^30^ Daubechies wavelets with 2 vanishing moments were used as a sparsity transform based on the code available from Lustig.^31^ Global low rank *r* was fixed for all experiments; LLR was determined as a fraction of the first singular value in each image block, i.e., $\lambda_{b}\propto S_{b}^{1}$, where $S_{b}^{1}$ is the first singular value at voxel *b* (note that the SVT of block *b* is determined by $\lambda_{b}$). The SLLR‐MRF reconstruction used the following parameters in simulations: rank *r* = 10, block size = 7, $\lambda_{b}$ = 0.03$S_{b}^{1}$, $\lambda_{w}$ **=** 0.01, $\mu_{1}$ = $\mu_{2}$ = 0.0005, ADMM iterations = 20, conjugate gradient iterations = 5. Phantom data were reconstructed with the same parameters, except $\lambda_{b}$ = 0.05$S_{b}^{1}$, $\mu_{1}$ = 0.01, $\lambda_{w}=\mu_{2}=0$ (i.e., no wavelet regularization). In vivo data at 2 × 2 mm^2^ and 1 × 1 mm^2^ resolutions used the following parameters, respectively: block size = 7 and 17, $\lambda_{b}$ = 0.05$S_{b}^{1}$ and 0.04$S_{b}^{1}$, $\lambda_{w}$ **=** 0.02 in both cases, $\mu_{1}$ = $\mu_{2}$ = 0.005 in both cases.

Reconstructions in simulations using only sparse (S‐LLR) or only LLR‐MRF regularizations were obtained by setting $\lambda_{b}=\mu_{1}=0$ or $\lambda_{w}=\mu_{2}=0$, respectively. A “warm start” strategy was used to improve the starting solution of subsequent ADMM iterations.^32^ Reconstruction parameters were empirically chosen by visually inspecting reconstructions from representative datasets, guided by previous literature. Rank *r* = 10 was chosen based on results from McGivney et al^11^; block size and $\lambda_{b}$ were chosen in a similar way to Zhang et al^20^; remaining parameters were determined empirically for simulations, phantom and in vivo data. The computational complexity of the proposed approach was dominated by 3 operations: (1) nonuniform fast Fourier transform^33^ (in solving Equation 5); (2) SVD (in solving Equation 6); (3) wavelet transform (in solving Equation 7). Solving Equation 5 has an estimated cost of $O(\left[aN+bN\log N\right]2rcN_{\mathrm{cg}})$, where a≈170 is related to gridding and interpolation costs, b≈2 is used for accuracy, N is the number of data points, r is the number of singular values, c is the number of coil channels and $N_{\mathrm{cg}}$ is the number of Conjugate gradient iterations; Equation 6 has an estimated cost of $O\left(s^{2}r^{2}N\right)$, where s is the block size for the locally low rank; Equation 7 has an estimated cost of O(2rN). The full estimated computational cost of the proposed approach is $O\left(\left\{\left[aN+bN\log N\right]2rcN_{\mathrm{cg}}+sr^{2}N+2rN\right\}N_{\mathrm{ADMM}}\right)$, where $N_{\mathrm{ADMM}}$ is the number of ADMM iterations.

In practice for this work, the bottleneck of operations was solving Equation 5, followed by solving Equation 6 and finally a negligible time to solve Equation 7. The reconstruction with 1750 time‐points took approximately 35 and 80 min (for 2 × 2 mm^2^ and 1 × 1 mm^2^, respectively) on a Linux workstation with 12 Intel Xeon X5675 (3.07 GHz) and 200 GB RAM. The 2 × 2 mm^2^ resolution data were reconstructed with (1750, 875, 584, 438) time‐point series. The 1 × 1 mm^2^ resolution data were reconstructed using the full 1750 time‐point series. For each case, images were reconstructed using the conventional low rank MRF approximation and the proposed SLLR‐MRF approach.

### 2.7 Dictionary and pattern recognition

MRF dictionaries were simulated using extended phase graphs based on the code available in Weigel.^34^ Slice profile correction,^35^ discretized into 50 points in the frequency dimension, was included. B_1_ inhomogeneity correction was not considered. The dictionary for the phantom acquisition was computed for the following sets of parameters: T_1_ ∈ [20:20:1600] ms, T_2_ ∈ [5:5:300] ms; the dictionary for the brain experiments (including simulations) used the following: T_1_ ∈ [0:10:800, 800:20:1400, 1400:100:6000] ms, T_2_ ∈ [0:1:100, 100:10:500, 500:20:1000, 1000:50:2600] ms.

## 3 RESULTS

### 3.1 Simulations

**Figure 2:**
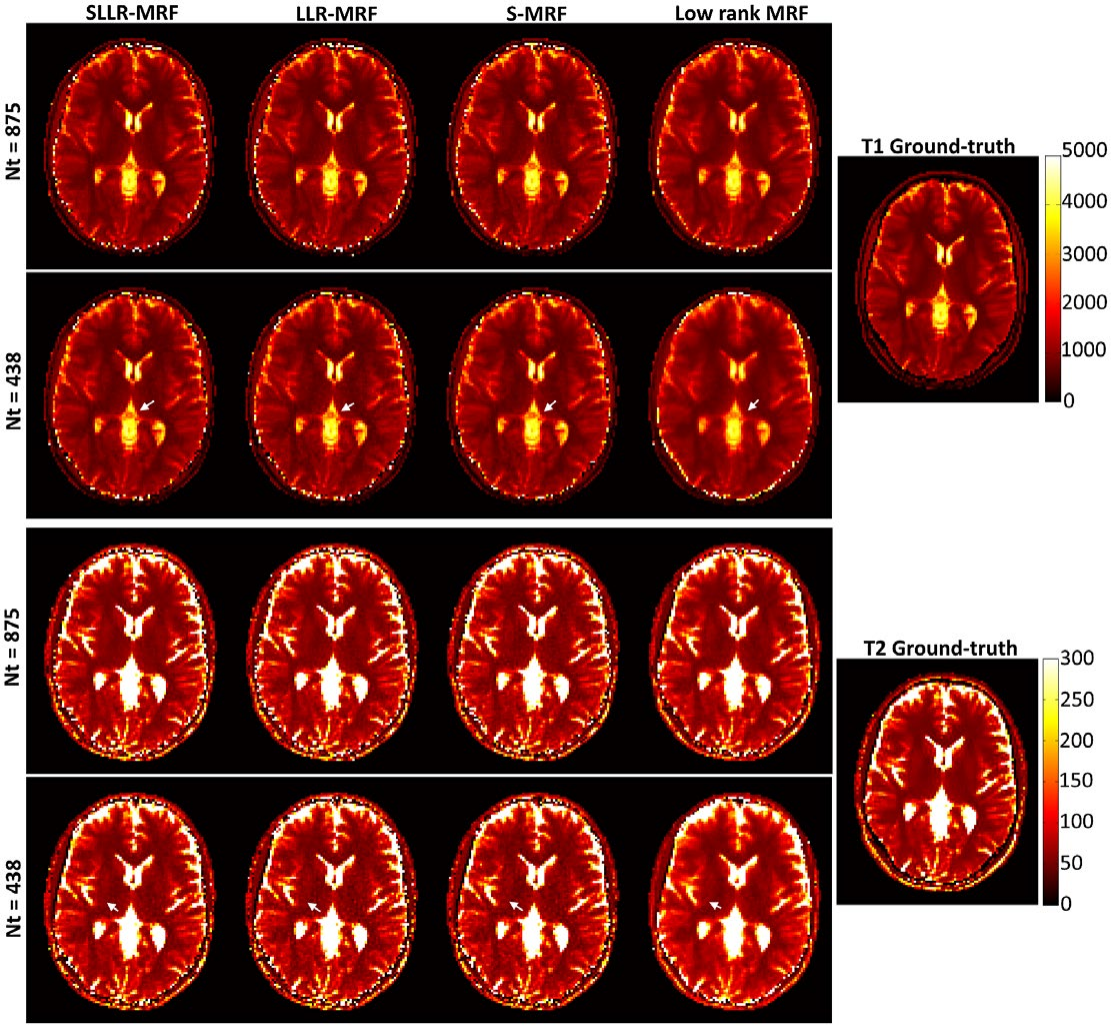
T_1_ and T_2_ parameter maps for different acceleration factors (Nt = 875 and Nt = 438), reconstructed with the proposed sparse and LLR constraints (SLLR‐MRF), only LLR constraint (LLR‐MRF), only sparse constraint (S‐MRF), and unconstrained low rank MRF, in simulations. All the constrained approaches use the same encoding operator as the low rank MRF, which enforces global low rank, compressing temporal time‐points into singular images. Comparable results are obtained with Nt = 875; however, appreciable differences exist with Nt = 438. Blurring and residual aliasing is present for low rank MRF; these are generally improved with S‐MRF and LLR‐MRF; the best quality maps are obtained with the proposed SLLR‐MRF

Parameter maps obtained in simulations with low rank MRF, S‐MRF, LLR‐MRF, and the proposed SLLR‐MRF using 875 and 438 time‐points are shown in Figure 2. Residual blurring and streaking artifacts present for low rank MRF at higher accelerations are reduced with S‐MRF, LLR‐MRF, and (more so) with SLLR‐MRF. Corresponding error maps are shown in Supporting Information Figure S1, which is available online, where this trend can be verified. NRMSE was measured for each of these reconstructions with 4 different acceleration factors, as shown in Table 1. Similar errors are observed with 1750 time‐points for all reconstructions, however, errors for the unconstrained low rank MRF increase faster than the constrained reconstructions (S‐MRF, LLR‐MRF, and SLLR‐MRF) as the acceleration factor increases, with SLLR‐MRF generally achieving the lowest errors.

**Table 1:** NRMSE for unconstrained low rank MRF, S‐MRF, LLR‐MRF and the proposed SLLR‐MRF, which includes both sparsity and LLR constraints, in simulations

NRMSE	R = 1	R = 2	R = 3	R = 4
	Nt = 1750	Nt = 875	Nt = 584	Nt = 438
T_1_ Low rank MRF	3.0%	5.2%	7.5%	9.0%
T_1_ S‐MRF	3.0%	5.0%	5.9%	6.2%
T_1_ LLR‐MRF	3.0%	4.3%	5.6%	6.2%
T_1_ SLLR‐MRF	**2.9**%	**3.9%**	**5.0%**	**5.4%**
T_2_ Low rank MRF	5.9%	10.0%	16.2%	18.1%
T_2_ S‐MRF	5.9%	9.1%	11.1%	12.8%
T_2_ LLR‐MRF	6.1%	8.8%	11.0%	13.2%
T_2_ SLLR‐MRF	**5.8%**	**8.0%**	**10.2%**	**11.4%**

Note

The values in boldface indicate the lowest NRMSE values among the methods compared.

**Figure 3:**
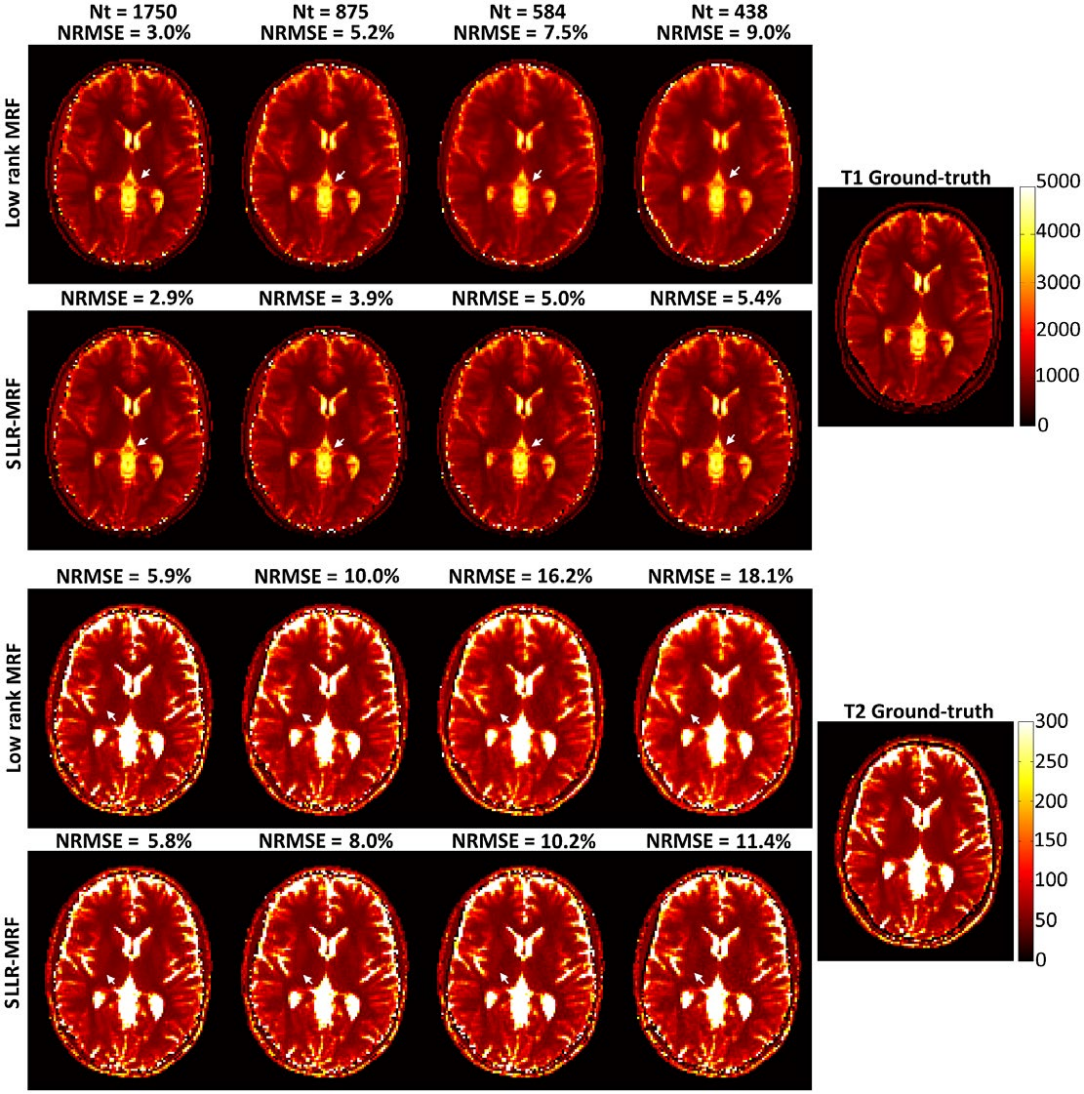
Reconstructed T_1_ and T_2_ maps using low rank MRF and the proposed SLLR‐MRF with different amounts of data for the simulation study. Residual blurring in T_1_ and residual aliasing in T_2_ increase as the time‐points are reduced with low rank MRF (white arrows); these artifacts are reduced with SLLR‐MRF. NRMSE increases with decreasing amount of data; however, the errors are reduced with the proposed approach

Parametric maps reconstructed with low rank MRF and SLLR‐MRF are shown in Figure 3 using different undersampling factors in simulations. As fewer time‐points are used, low rank MRF gradually introduces blurring in T_1_ and residual aliasing in T_2_ maps; these artifacts are considerably reduced with the proposed SLLR‐MRF. Corresponding error maps are shown in Supporting Information Figure S2, where lower errors were generally obtained with SLLR‐MRF. Simulations generally showed higher errors in T_2_. Selected reconstructed time‐point images corresponding to these maps are shown in Supporting Information Figure S3, where a considerable reduction in aliasing artifacts can be observed using the proposed SLLR‐MRF approach. Indeed, some of these artifacts propagate into parameter maps, as indicated by the arrows in Figure 3.

### 3.2 Data acquisition

#### 3.2.1 Phantom study

**Figure 4:**
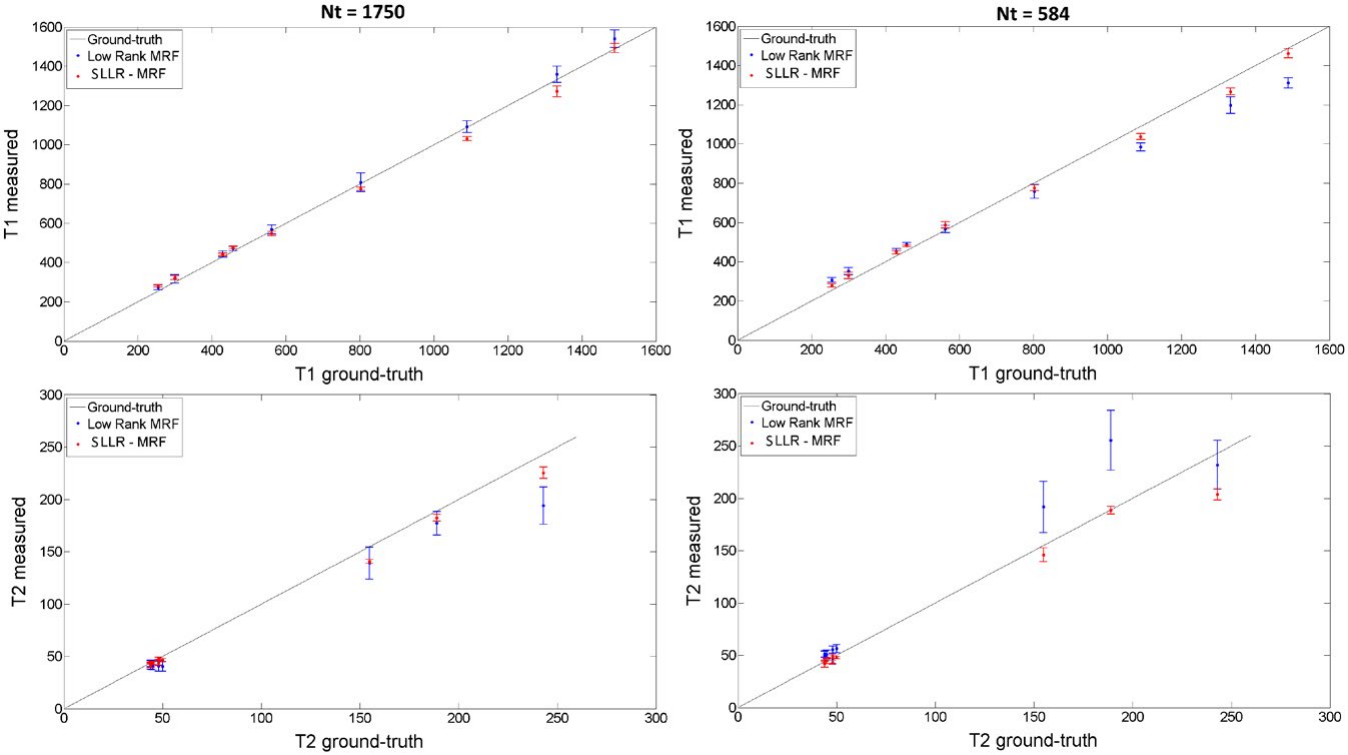
Plots for T_1_ and T_2_ values using low rank MRF and SLLR‐MRF with 1750 and 584 time‐points for the phantom study. Higher accuracy is generally achieved with the proposed method. As the number of time‐points is reduced, accuracy and precision of low rank MRF are reduced, particularly for T_2_. Conversely, accuracy and precision are maintained with SLLR‐MRF when the number of time‐points is reduced to 584

Plots for T_1_ and T_2_ measurements in the phantom can be seen in Figure 4 for 1750 and 584 time‐points in comparison to the gold standard values provided by the vendor.^29^ Similar accuracy is achieved with both low rank MRF and SLLR‐MRF for 1750 time‐points; however, SLLR‐MRF leads to improved precision. When the number of time‐points is reduced to 584 precision and accuracy are reduced with low rank MRF, whereas SLLR‐MRF performs similarly to the case with 1750 time‐points. Corresponding bias and standard deviations of the phantom measurements are listed in Table 2, where reduced bias and standard deviation is generally obtained with SLLR‐MRF (particularly at higher accelerations). Underestimation of T_1_ and T_2_ for high values can be observed for SLLR‐MRF; bias of low rank MRF varied with the number of time‐points due to the induced aliasing artifacts. Corresponding parametric maps are shown in Supporting Information Figure S4, where SLLR‐MRF with 582 time‐points achieves similar quality to low rank MRF with 1750 time‐points.

**Table 2:** Bias and standard deviation for each phantom tube corresponding to Figure 4 and Supporting Information Figure S4

Tube	Nt = 1750		Nt = 584		Ground‐Truth
	Low rank MRF	SLLR‐MRF	Low rank MRF	SLLR‐MRF	
T1	Bias ± SD	Bias ± SD	Bias ± SD	Bias ± SD	T1
A	13 ± 17	10 ± 6	23 ± 14	17 ± 9	430
B	7 ± 23	−14 ± 12	4 ± 19	24 ± 16	562
C	17 ± 22	24 ± 10	52 ± 18	29 ± 17	300
D	2 ± 31	−59 ± 11	−106 ± 20	−52 ± 15	1090
E	26 ± 41	−61 ± 28	−135 ± 43	−66 ± 17	1333
F	6 ± 47	−27 ± 8	−45 ± 35	−27 ± 15	803
G	13 ± 11	19 ± 7	30 ± 10	23 ± 4	458
H	51 ± 45	4 ± 24	−178 ± 27	−27 ± 23	1489
I	18 ± 12	26 ± 6	52 ± 13	24 ± 8	255
T2	Bias ± SD	Bias ± SD	Bias ± SD	Bias ± SD	T2
A	−0.7 ± 3.2	0.2 ± 0.4	7.1 ± 3.1	0.4 ± 0.5	44
B	−3.9 ± 4.0	−1.3 ± 1.6	5.6 ± 4.2	−0.2 ± 2.7	45
C	−2.2 ± 4.1	−2.6 ± 1.9	5.5 ± 4.3	−2.0 ± 3.4	44
D	−13.7 ± 2.8	−10.0 ± 1.4	−2.6 ± 3.5	−9.2 ± 1.8	58
E	−9.6 ± 4.4	−3.3 ± 1.6	6.3 ± 3.9	−2.3 ± 1.2	50
F	−17.2 ± 4.7	−14.6 ± 2.3	−10.9 ± 4.9	−14.8 ± 1.9	58
G	−11.7 ± 11.2	−6.5 ± 3.3	66.4 ± 28.6	−0.4 ± 3.8	189
H	−48.9 ± 17.7	−17.5 ± 5.6	−11.1 ± 23.5	−39.4 ± 5.3	243
I	−15.8 ± 15.4	−14.2 ± 1.8	36.7 ± 24.4	−9.1 ± 6.7	155

Note

Generally, smaller biases and standard deviations are obtained with SLLR‐MRF, particularly for higher acceleration (Nt = 584).

#### 3.2.2 In vivo study

**Figure 5:**
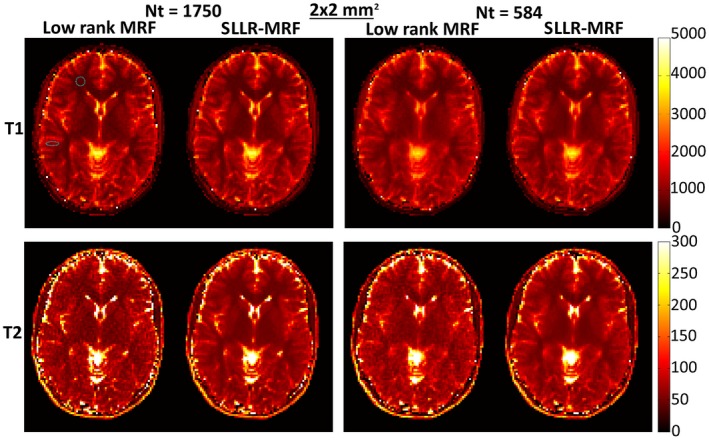
T_1_ and T_2_ maps using low rank MRF and the proposed SLLR‐MRF with different amounts of data, for subject 1, 2 × 2 mm^2^ resolution in vivo. Residual blurring in T_1_ and noise amplification in T_2_ are present for low rank MRF, particularly when only 584 time‐points are used. SLLR‐MRF maintains similar parametric map quality even with a reduced number of time‐points. The dotted circles denote the regions of interest used to measure T_1_ and T_2_ values for white and gray matter, collected in Table 3

**Figure 6:**
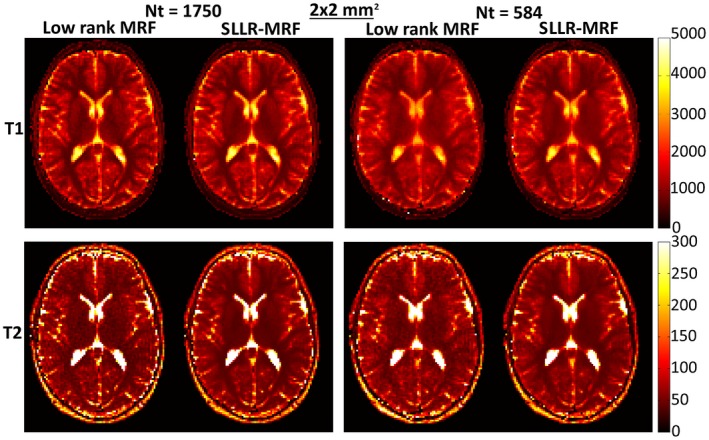
T_1_ and T_2_ maps using low rank MRF and the proposed SLLR‐MRF with different amounts of data, for subject 2, 2 × 2 mm^2^ resolution in vivo. Similar to Figure 5, SLLR‐MRF maintains parametric map quality at reduced number of time‐points, whereas blurring and noise amplification is present with low rank MRF

Low rank MRF and SLLR‐MRF parametric maps for 2 representative healthy subjects at 2 × 2 mm^2^ resolution, using different lengths of the time‐point series (1750 and 584), are shown in Figures 5 and 6. Residual blurring artifacts in T_1_ and noise amplification in T_2_ can be seen with low rank MRF, which increase with decreasing number of time‐points. SLLR‐MRF improves the parametric map quality in every case and maintains good map quality even when only a third of time‐points are used. Corresponding reconstructed time‐point images are shown in Supporting Information Figure S5 and S6. In these figures, time‐points with high T_1_ and T_2_ encoding power are shown (#100 and #1600, respectively). Low rank MRF has residual artifacts when the number of time‐points is reduced, whereas these are considerably reduced with the proposed SLLR‐MRF.

**Figure 7:**
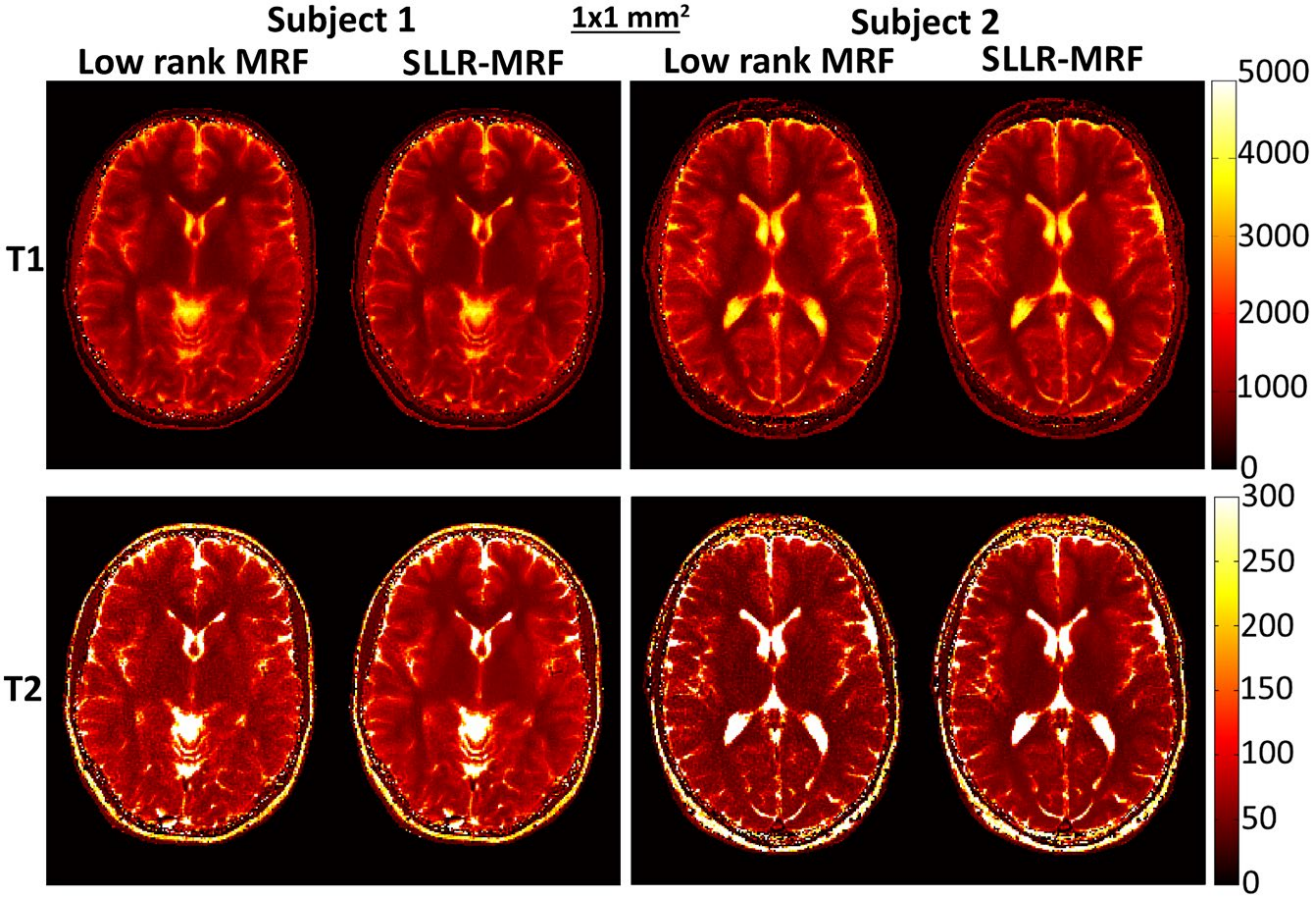
T_1_ and T_2_ maps using low rank MRF and the proposed SLLR‐MRF with different amounts of data, for subjects 1 and 2, 1 × 1 mm^2^ resolution in vivo using 1750 time‐points. Minimal blurring is present in T_1_ for low rank MRF; however, considerable noise amplification is present in T_2_ with low rank MRF, which is reduced with SLLR‐MRF

Low rank MRF and SLLR‐MRF parametric maps for corresponding subjects at 1 × 1 mm^2^ resolution can be seen in Figure 7. Minimal blurring was observed in T_1_ for this case, but noise amplification was once again observed with low rank MRF and reduced with SLLR‐MRF. Corresponding time‐point images are shown in Supporting Information Figure S7, where reconstructions with low rank MRF contain considerable more residual aliasing than SLLR‐MRF.

T_1_ and T_2_ values in selected regions on interest (marked in Figure 5) for white matter and gray matter are shown in Table 3 for all in vivo experiments. Consistent qualitative results between different methods and resolutions were generally achieved; however, slightly increased values were observed for T_2_ at 1 × 1 mm^2^ compared with 2 × 2 mm^2^ resolution.

**Table 3:** T_1_ and T_2_ values in white and gray matter measured on the corresponding regions of interest in Figure 5

	2 × 2 mm^2^ dataset		1 × 1 mm^2^ dataset		Literature
	Low rank MRF	SLLR‐MRF	Low rank MRF	SLLR‐MRF	
	Nt = 1750	Nt = 584	Nt = 1750	Nt = 1750	
White matter T_1_	659 ± 35	676 ± 44	672 ± 34	704 ± 35	608–756
Grey matter T_1_	1111 ± 42	1108 ± 48	1089 ± 58	1064 ± 51	998–1304
White matter T_2_	45.5 ± 6.4	44.6 ± 6.1	49.5 ± 6.8	46.7 ± 6.3	54–81
Grey matter T_2_	66.1 ± 6.2	65.5 ± 7.5	65.7 ± 9.5	65.8 ± 9.5	78–98

Note.

Values shown correspond to the mean ± standard deviation of the parametric values measured across the five healthy subjects.

## 4 DISCUSSION

In this work, we study the use of sparsity and locally low rank constraints for accelerated MRF, combining it with the previously proposed low rank approximation. Results in simulations, standardized phantom, and in vivo acquisitions indicate that the proposed SLLR‐MRF enables accurate parametric mapping at higher undersampling factors and/or lower SNR levels than using only a low rank approximation.

Aliasing and noise amplification in the reconstructed singular images (and thus in the time‐point series) can propagate into the MRF parametric maps. Low rank MRF results in simulations indicate that residual artifacts from radial sampling generally lead to blurring and noise amplification in the reconstructed T_1_ and T_2_ maps, respectively, for high acceleration factors (i.e., reduced number of time‐point images, with 1 radial spoke per time‐point). SLLR‐MRF considerably reduced residual aliasing, improving parametric map quality. Simulation results agreed with a T_1_/T_2_ phantom acquisition showing superior accuracy and precision for SLLR‐MRF in comparison to low rank MRF for different lengths of the time‐point series. A slight underestimation of T_2_ was generally observed in the phantom experiments, more so at high T_2_ values. A small difference in T_2_ measurements was also observed in vivo between 2 × 2 mm^2^ and 1 × 1 mm^2^ resolution. These errors can be reduced by incorporating B_1_ correction,^36^ with improved FA patterns and readouts,^37^, ^38^ magnetization transfer correction,^39^ and partial volume correction.^40^, ^41^ A slight underestimation for high T_1_ values was also observed; this bias could also be reduced by accounting for magnetization transfer and inversion efficiency.^42^ These corrections were not considered in this study and will need to be investigated in future studies.

The large slice thickness (10 mm) used is also a limitation in this study. Thinner slices (in the order of 5 mm) are generally desirable and will have a corresponding reduction in SNR. Sparse and low rank constraints are generally well suited to suppress noise‐like artifacts, although this problem can be complementarily addressed in the acquisition. Increasing the amount of acquired data or reducing the receiver bandwidth will both improve SNR at the expense of scan time. A more data efficient trajectory such as spiral can lead to SNR improvements with no scan time penalty. Improved SNR can be achieved at 3T; however, B_0_ and B_1_ corrections become increasingly necessary with increasing field strength. If B_0_ is accounted for in the MRF model (or if field inhomogeneities are minimal) bSSFP MRF can be used to achieve superior SNR in similar scan time. Finally, recent studies have revealed optimal MRF sequences^37^, ^38^ that lead to improved SNR in the corresponding parameter maps.

In vivo scans with 2 × 2 mm^2^ in‐plane resolution showed that both low rank MRF approximation and SLLR‐MRF achieved good map quality with 1750 time‐point images. However, a reduction of the number of time points resulted in decreased quality for the low rank MRF reconstruction. Conversely, with SLLR‐MRF the number of time‐points could be reduced by a third (i.e., from 7.5 s scan time to ~2.6 s) while maintaining good parametric map quality. Retrospective uniform undersampling in time by keeping (1:*n*:1750), with n = 1, 2, 3, 4, was used here to approximate a similar encoding of T_1_ and T_2_ values for experiments with different amounts of data. If only the first time‐point images were considered the influence of the initial inversion pulse and the reduced number of FA values would result in different T_1_ and T_2_ encodings that those achieved by longer scans, and thus direct comparison between acquisitions would not be adequate.

The better performance of SLLR‐MRF, demonstrated in simulations, phantom, and in vivo scans, was used here to enable in vivo acquisitions with higher in‐plane spatial resolution (and thus higher undersampling factor and lower SNR). Acquisitions were performed with 1 × 1 mm^2^ spatial resolution and the same amount of data (1750 time‐points), thus comparable scan time, as the 2 × 2 mm^2^ maps. T_2_ noise amplification was considerable with low rank MRF and consequently reduced with SLLR‐MRF. Optimal FA patterns^37^, ^38^ need to be investigated in the future to ensure sufficient T_1_ and T_2_ encoding for 2D MRF with highly reduced scan time. Moreover, the advantages of the proposed approach should be more evident in 3D acquisitions where higher undersampling factors are required. The extension of the proposed method to 3D MRF^43^ will be investigated in future studies.

The proposed SLLR‐MRF is expected to be sensitive to motion, as previously observed in other MRF studies.^44^, ^45^, ^46^, ^47^ Due to the global rank constraint within the encoding operator of the SLLR‐MRF, each singular image is (potentially) a linear combination of every time‐point. Consequently, stronger motion artifacts are expected when a global low rank reconstruction is used, when compared with a zero‐filled or a sliding window reconstruction. This limitation can be addressed with motion correction techniques, which is a problem currently under investigation.^44^, ^45^, ^46^, ^47^

Another limitation of the proposed approach is related to parameter selection. Similar to most MRI reconstruction methods, the proposed approach has several tuneable parameters (rank, block size, $\lambda_{b}$, $\lambda_{w}$, $\mu_{i}$, ADMM iterations, conjugate gradient iterations) that will affect the reconstruction if improperly chosen. The same parameters were used for all the results reported here; however, future studies in larger cohort of subjects need to be performed to investigate the sensitivity of the chosen parameters. The proposed approach was compared against a ground‐truth in simulations and phantom experiments; however, comparison with a fully sampled MRF in vivo was not possible due to scan time constraints. Current reconstruction times were in the order of 1 h for high in‐plane resolution data; this will need to be reduced in the future with implementations in more efficient compiled languages (e.g., C++) and/or graphical processing units.

## 5 CONCLUSIONS

A sparsity and LLR regularization for the low rank approximation reconstruction in MRF has been introduced and validated in simulations, standardized phantom, and in vivo brain acquisitions. The proposed SLLR‐MRF approach removed blurring in T_1_ and noise amplification in T_2_ observed in the unregularized low rank MRF approximation. SLLR‐MRF enabled a reduction of time‐points from 1750 to ~600 (potentially reducing scan time from 7.5 s to ~2.6 s) while maintaining map quality in 2 × 2 mm^2^ in‐plane resolution data. SLLR‐MRF enabled a considerable improvement in parametric map quality compared with low rank MRF in 1 × 1 mm^2^ resolution data acquired in 9.6 s.

## Supporting information


**FIGURE S1** T_1_ and T_2_ error maps (in milliseconds), for the corresponding parameter maps in Figure 2, reconstructed with the proposed sparse and local low rank constraints (SLLR‐MRF), only local low rank constraint (LLR‐MRF), only sparse constraint (S‐MRF) and unconstrained low rank MRF. Skull and CSF have been masked out when computing errors. Error maps correlate with the parameter maps shown in Figure 2, with higher errors obtained for the low rank MRF and the lowest errors obtained for the proposed SLLR‐MRF
**FIGURE S2** T_1_ and T_2_ error maps (in milliseconds), for the corresponding parameter maps in Figure 3, reconstructed with unconstrained low rank MRF and the proposed SLLR‐MRF. A mask has been used to exclude skull and CSF tissue in the error maps. Errors gradually increase with increasing acceleration factor (decreasing Nt) for both approaches, however, errors are generally milder for the proposed SLLR‐MRF. Corresponding normalized root mean square errors (NRMSE) for these maps can be found in Table 1
**FIGURE S3** Reconstructed time points #100 and #1600 reconstructed with low rank MRF and the proposed SLLR‐MRF in simulations. Both methods achieve similar time‐point image quality with 1750 time‐points. Aliasing artifacts appear in low rank MRF when the number of time‐points is reduced; these artifacts are considerably reduced with SLLR‐MRF
**FIGURE S4** T1 and T2 maps for a standardized phantom reconstructed with low rank MRF and the proposed SLLR‐MRF with 1750 and 584 time‐points. Larger errors are generally present with low rank MRF, more so when less data is used. When using 584 time‐points, the proposed SLLR‐MRF achieves similar quality to the low rank MRF with 1750 time‐points
**FIGURE S5** Time points #100 and #1600 for low rank MRF and the proposed SLLR‐MRF, reconstructed using 1750 and 584 total number of time‐points, for subject 1, 2 × 2 mm^2^ resolution. Residual aliasing is visible for low rank MRF when the number of time‐points is reduced. Conversely, these artifacts are reduced with the proposed SLLR‐MRF
**FIGURE S6** Time points #100 and #1600 for low rank MRF and the proposed SLLR‐MRF, reconstructed using 1750 and 584 total number of time‐points, for subject 2, 2 × 2 mm^2^ resolution. Residual aliasing is visible for low rank MRF when the number of time‐points is reduced. Conversely, these artifacts are reduced with the proposed SLLR‐MRF
**FIGURE S7** Time points #100 and #1600 for low rank MRF and the proposed SLLR‐MRF, reconstructed using 1750 and 584 total number of time‐points, for subjects 1 and 2, 1 × 1 mm^2^ resolution. Residual aliasing is present with low rank MRF, whereas SLLR‐MRF reduces aliasing artifacts
