# Supplementary material for: Sparsity and locally low rank regularization for MR fingerprinting
Source: Magn Reson Med. 2019 Feb 5;81(6):3530–43. doi: 10.1002/mrm.27665 (PMC6492150; doi:10.1002/mrm.27665)
Supplement: Supplementary file 1 — FIGURE S1 T1 and T2 error maps (in milliseconds), for the corresponding parameter maps in Figure 2, reconstructed with the proposed sparse and local low rank constraints (SLLR‐MRF), only local low rank constraint (LLR‐MRF), only sparse constraint (S‐MRF) and unconstrained low rank MRF. Skull and CSF have been masked out when computing errors. Error maps correlate with the parameter maps shown in Figure 2, with higher errors obtained for the low rank MRF and the lowest errors obtained for the proposed SLLR‐MRF FIGURE S2 T1 and T2 error maps (in milliseconds), for the corresponding parameter maps in Figure 3, reconstructed with unconstrained low rank MRF and the proposed SLLR‐MRF. A mask has been used to exclude skull and CSF tissue in the error maps. Errors gradually increase with increasing acceleration factor (decreasing Nt) for both approaches, however, errors are generally milder for the proposed SLLR‐MRF. Corresponding normalized root mean square errors (NRMSE) for these maps can be found in Table 1 FIGURE S3 Reconstructed time points #100 and #1600 reconstructed with low rank MRF and the proposed SLLR‐MRF in simulations. Both methods achieve similar time‐point image quality with 1750 time‐points. Aliasing artifacts appear in low rank MRF when the number of time‐points is reduced; these artifacts are considerably reduced with SLLR‐MRF FIGURE S4 T1 and T2 maps for a standardized phantom reconstructed with low rank MRF and the proposed SLLR‐MRF with 1750 and 584 time‐points. Larger errors are generally present with low rank MRF, more so when less data is used. When using 584 time‐points, the proposed SLLR‐MRF achieves similar quality to the low rank MRF with 1750 time‐points FIGURE S5 Time points #100 and #1600 for low rank MRF and the proposed SLLR‐MRF, reconstructed using 1750 and 584 total number of time‐points, for subject 1, 2 × 2 mm2 resolution. Residual aliasing is visible for low rank MRF when the number of time‐points is reduced. Conversely, these artifac [file MRM-81-3530-s001.docx]

**Supporting Information:**


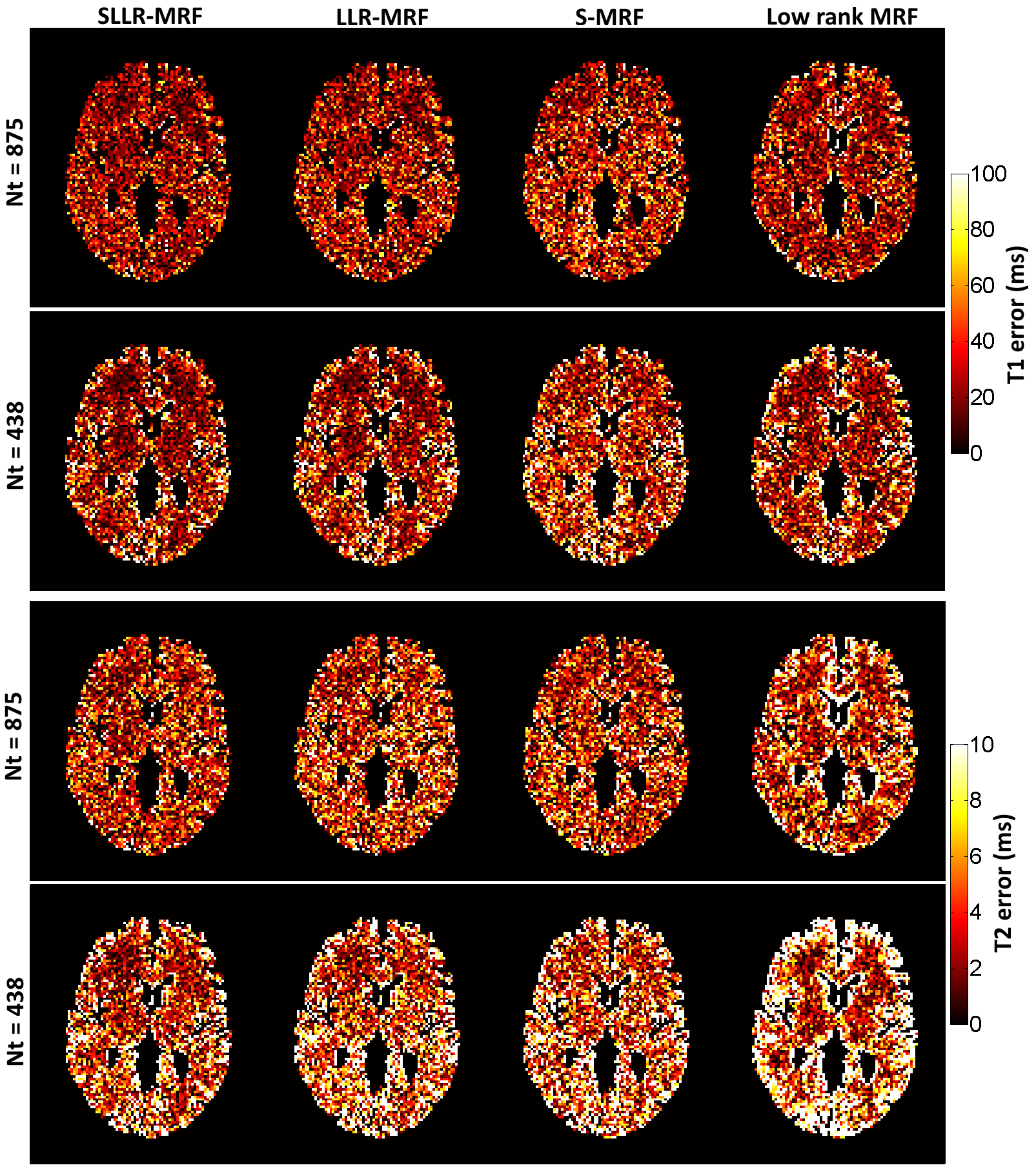


Supporting Information Figure S1. T_1_ and T_2_ error maps (in milliseconds), for the corresponding parameter maps in Figure 2, reconstructed with the proposed sparse and local low rank constraints (SLLR-MRF), only local low rank constraint (LLR-MRF), only sparse constraint (S-MRF) and unconstrained low rank MRF. Skull and CSF have been masked out when computing errors. Error maps correlate with the parameter maps shown in Figure 2, with higher errors obtained for the low rank MRF and the lowest errors obtained for the proposed SLLR-MRF.


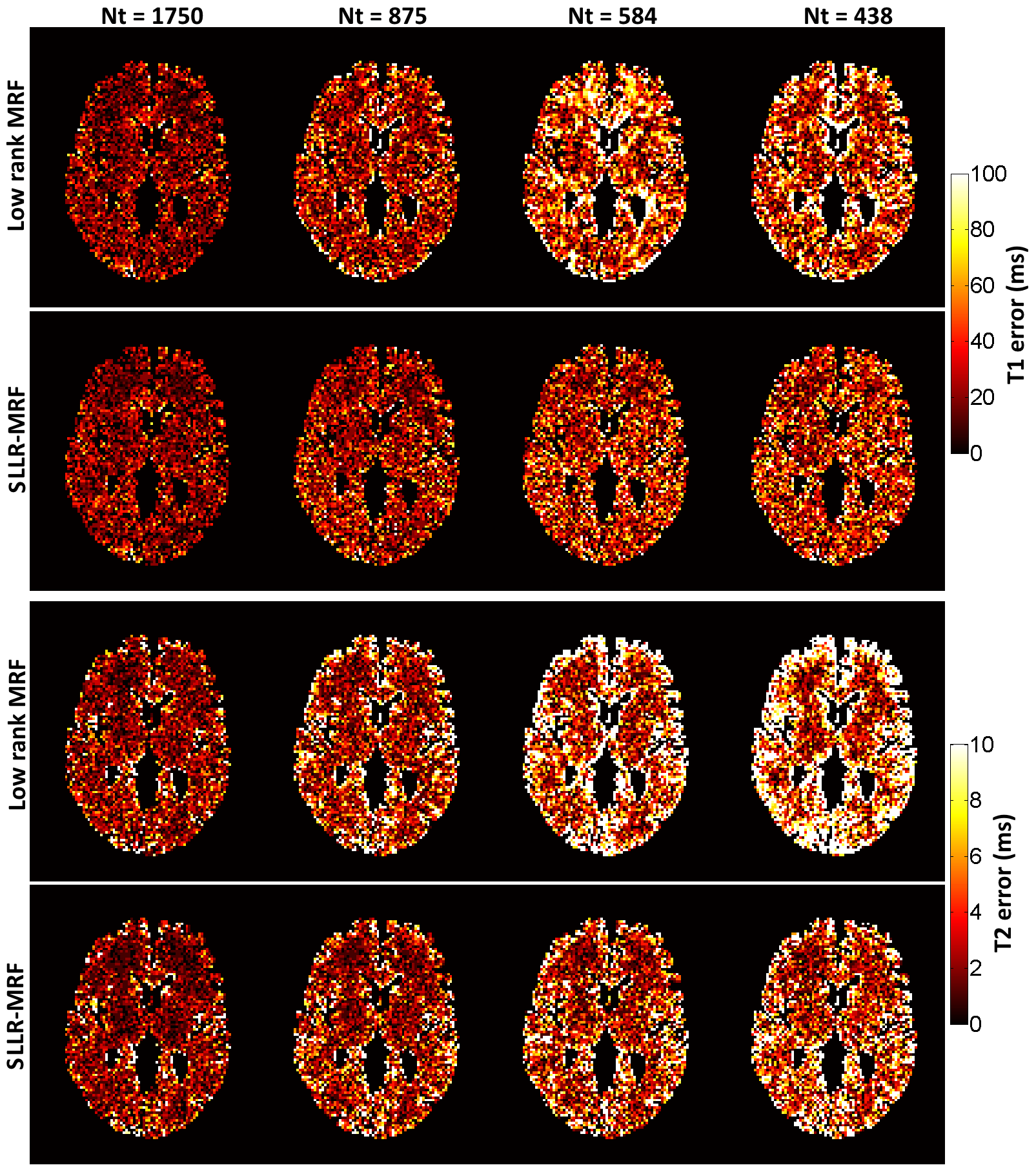


Supporting Information Figure S2. T_1_ and T_2_ error maps (in milliseconds), for the corresponding parameter maps in Figure 3, reconstructed with unconstrained low rank MRF and the proposed SLLR-MRF. A mask has been used to exclude skull and CSF tissue in the error maps. Errors gradually increase with increasing acceleration factor (decreasing Nt) for both approaches, however errors are generally milder for the proposed SLLR-MRF. Corresponding normalized root mean square errors (NRMSE) for these maps can be found in Table 1.


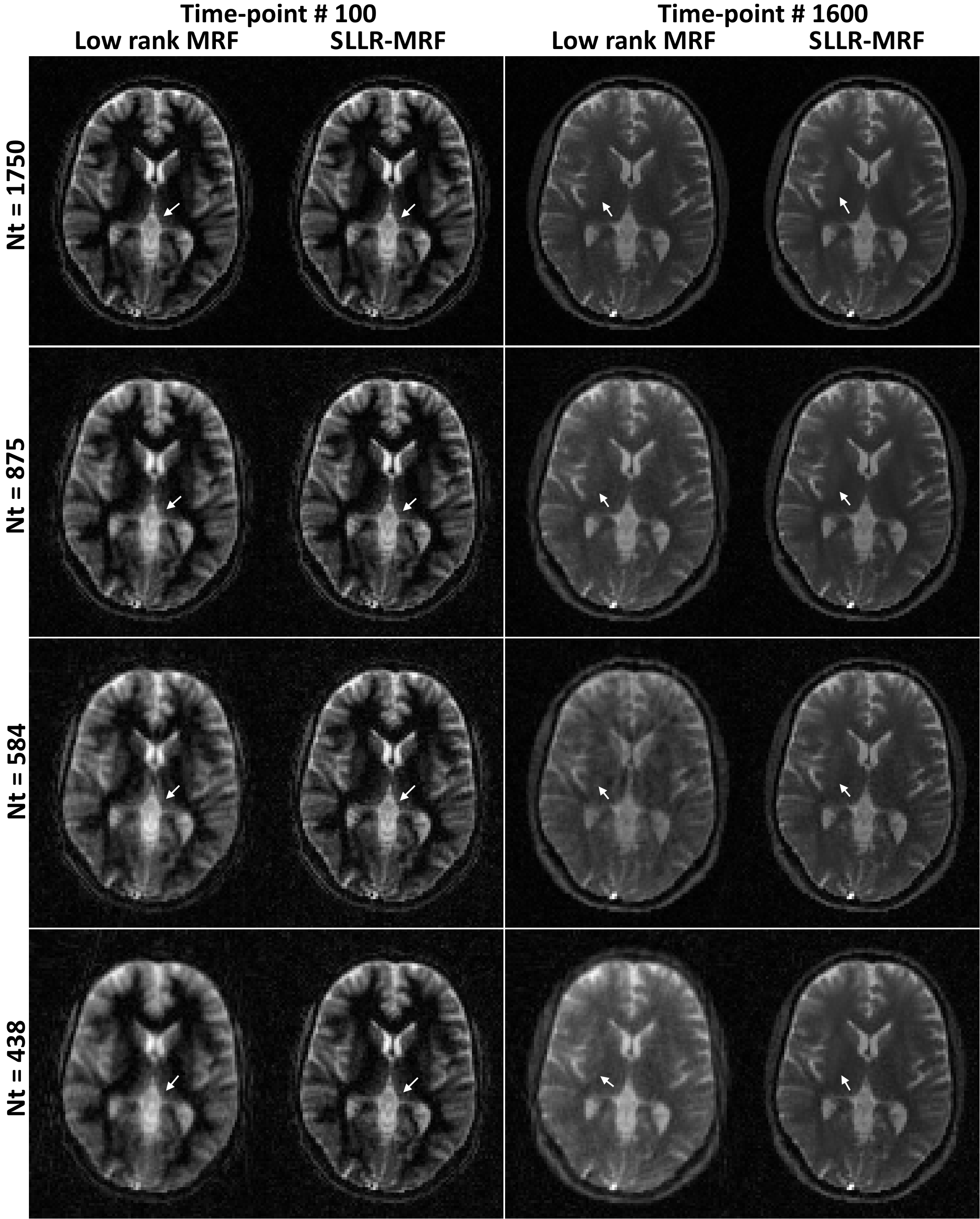


Supporting Information Figure S3: Reconstructed time points #100 and #1600 reconstructed with low rank MRF and the proposed SLLR-MRF in simulations. Both methods achieve similar time-point image quality with 1750 time-points. Aliasing artefacts appear in low rank MRF when the number of time-points is reduced; these artefacts are considerably reduced with SLLR-MRF.


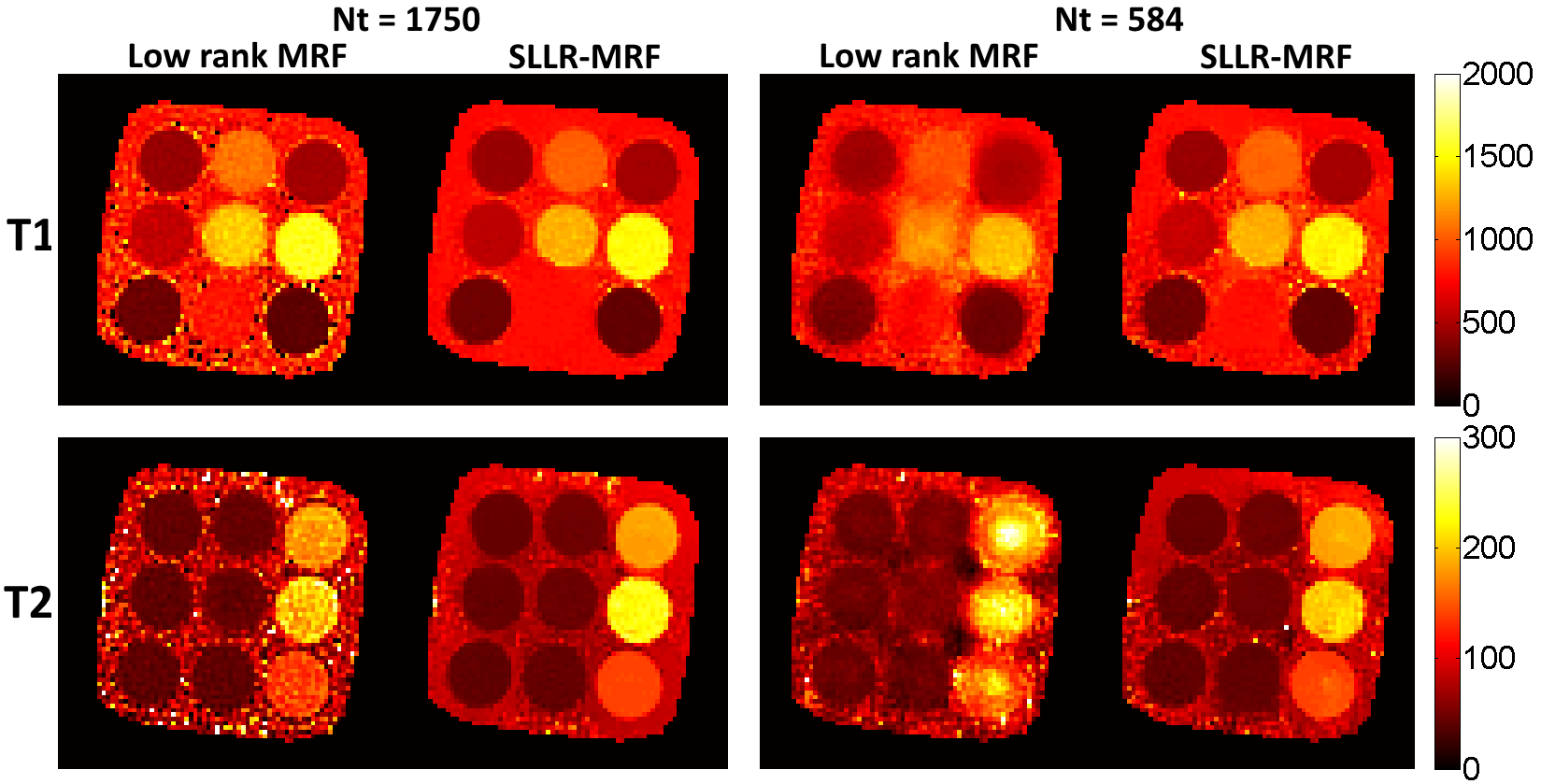


Supporting Information Figure S4: T_1_ and T_2_ maps for a standardized phantom reconstructed with low rank MRF and the proposed SLLR-MRF with 1750 and 584 time-points. Larger errors are generally present with low rank MRF, more so when less data is used. When using 584 time-points, the proposed SLLR-MRF achieves similar quality to the low rank MRF with 1750 time-points.


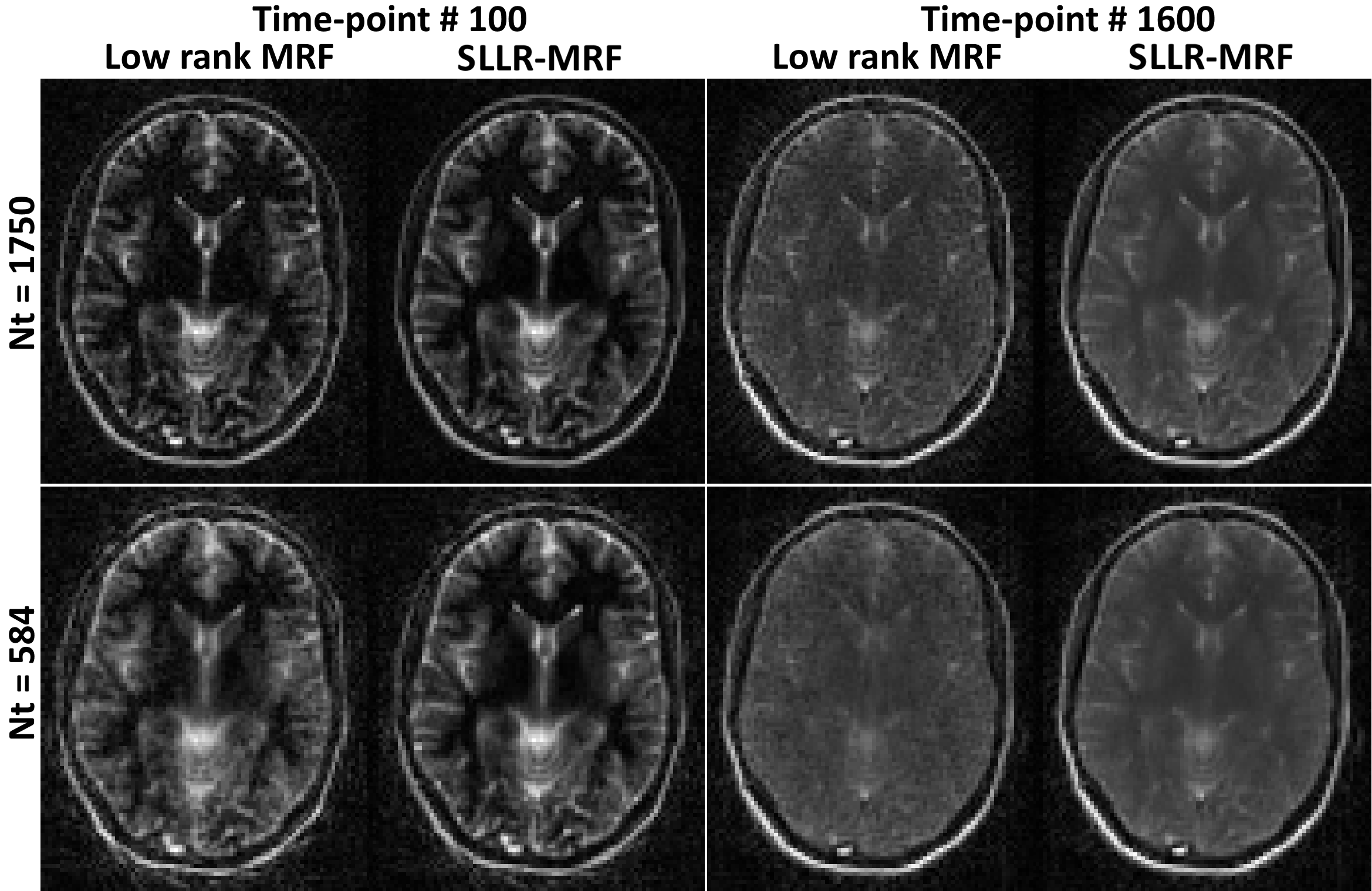


Supporting Information Figure S5: Time points #100 and #1600 for low rank MRF and the proposed SLLR-MRF, reconstructed using 1750 and 584 total number of time-points, for subject 1, 2x2 mm^2^ resolution. Residual aliasing is visible for low rank MRF when the number of time-points is reduced. Conversely, these artefacts are reduced with the proposed SLLR-MRF.


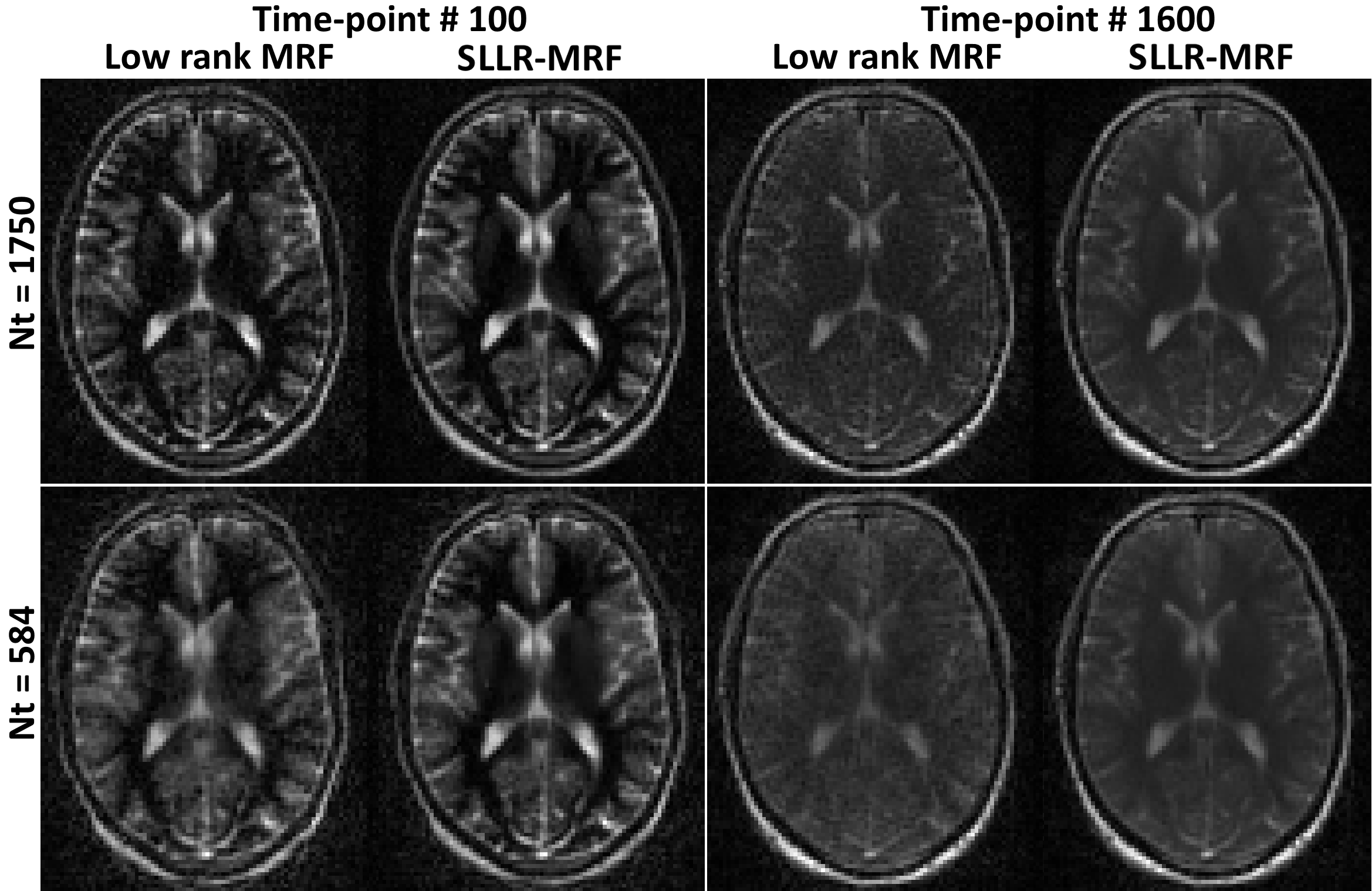


Supporting Information Figure S6: Time points #100 and #1600 for low rank MRF and the proposed SLLR-MRF, reconstructed using 1750 and 584 total number of time-points, for subject 2, 2x2 mm^2^ resolution. Residual aliasing is visible for low rank MRF when the number of time-points is reduced. Conversely, these artefacts are reduced with the proposed SLLR-MRF.


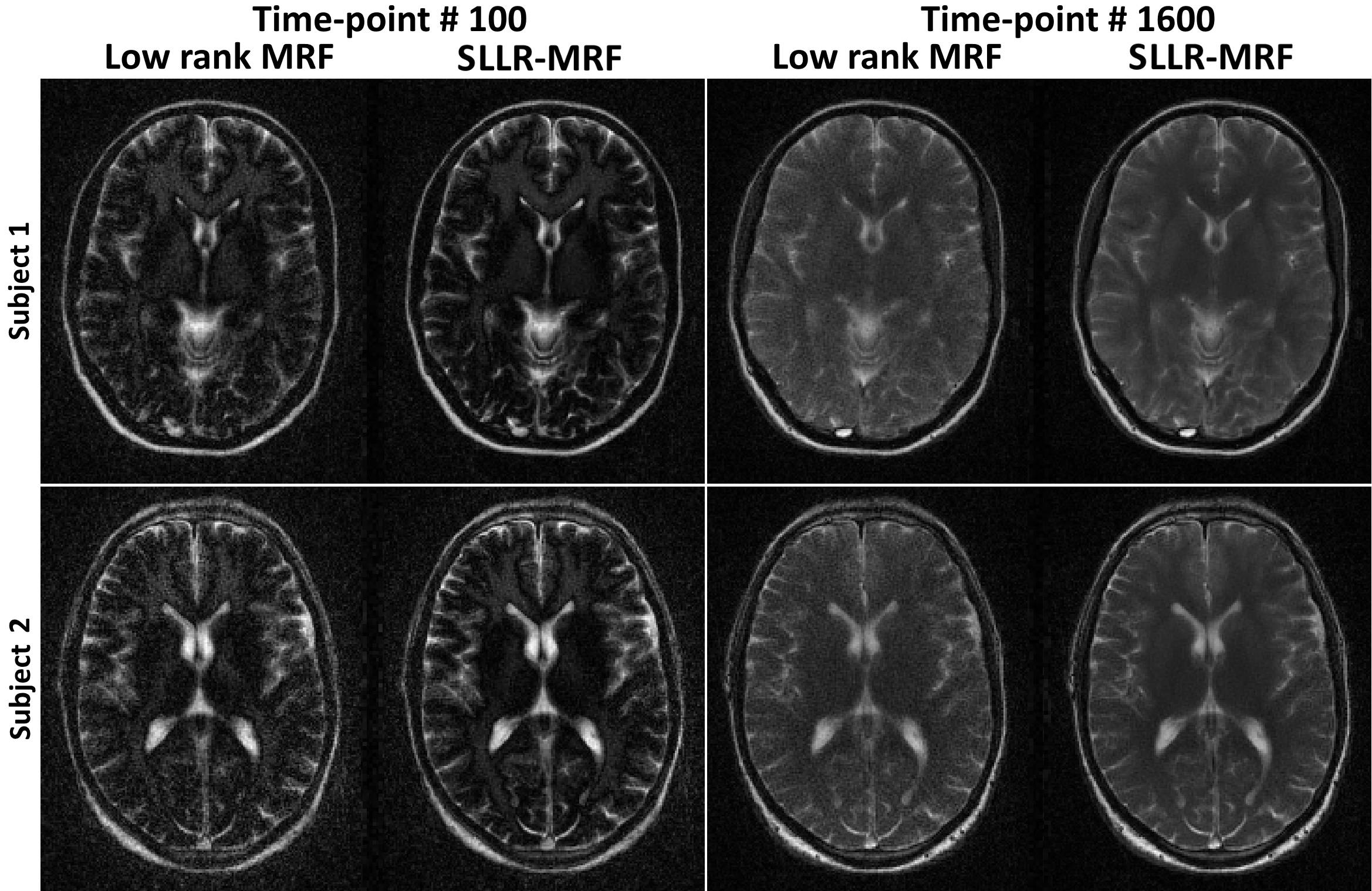


Supporting Information Figure S7: Time points #100 and #1600 for low rank MRF and the proposed SLLR-MRF, reconstructed using 1750 and 584 total number of time-points, for subjects 1 and 2, 1x1 mm^2^ resolution. Residual aliasing is present with low rank MRF, whereas SLLR-MRF reduces aliasing artefacts.
